# Trajectories of second language student classroom engagement: Profiles and correlates

**DOI:** 10.1111/bjep.70081

**Published:** 2026-04-06

**Authors:** Hoi Vo

**Affiliations:** ^1^ Faculty of Education Southern Cross University Bilinga Queensland Australia

**Keywords:** growth mixture modelling, growth trajectories, positive and negative classroom affect, second language classroom engagement, teacher emotional support

## Abstract

**Background:**

Research on the development of second language (L2) student classroom engagement is growing; however, this body of research primarily relies on designs that preclude the examination of whether this development is common to all students or whether different developmental trajectories emerge for different groups.

**Aim:**

Adopting a person‐centred longitudinal approach and using growth mixture modelling, this study aims to discover distinct groups of students with varied trajectories of L2 classroom engagement over their first semester at university and to examine how these profiles of engagement trajectories shape and are shaped by classroom dynamics.

**Sample:**

Data were collected from 497 English as a foreign language learners in Vietnam across three time points over a 10‐week semester.

**Method:**

A person‐centred longitudinal design was employed, with growth mixture modelling used to identify distinct engagement trajectory groups and to examine their associations with classroom dynamics.

**Results:**

Four distinct groups of engagement trajectories were identified: a high initial–slight increase, a high initial–stable, a moderate initial–slight decrease and a moderate initial–steep decrease group. Students who reported higher levels of perceived teacher sensitivity were more likely to be in the high initial–slight increase group than in any other groups, whereas students in the moderate initial–slight decrease group showed the lowest levels of positive classroom affect and the highest levels of negative classroom affect.

**Conclusion:**

The findings highlight the importance of designing engagement support programs tailored to the needs of different groups of students, recognising the varied developmental trajectories of L2 student classroom engagement.

## INTRODUCTION

Language learning is not simply a transactional process. It requires the devotion of physical, mental and psychological energy and resources on the part of the learners because, for language learning to be successful, learners need to be actively involved in the practice and use of the language over an extended period of time. Therefore, understanding the nature of learner engagement and what fosters such engagement in instructed second language learning contexts is key to successful language learning. The past 10 years have witnessed an exponential growth in the number of second language (L2) learner engagement studies, contributing to a richer understanding of what L2 engagement entails, how it is shaped by multilayered contextual influences and the implications it has for second language development (Hiver et al., 2021). As there is a steady increase in studies that reinforce theoretical and conceptual understanding of the nature of L2 learner engagement, particularly its situated, dynamic and malleable characteristics, so too is the need for empirical research that looks at the development of L2 engagement over time and what triggers or sustains such development (Vo et al., 2024). In response to this, an emerging body of longitudinal L2 engagement research has started to explore how L2 learner engagement fluctuates across contexts and timescales. For example, L2 engagement has been shown to fluctuate considerably over the course of a task (Aubrey et al., 2020; Dao & Sato, 2021), a lesson (Sulis, 2022) or a semester (Vo et al., 2024; Zhou, Hiver, & Zheng, 2023). Such fluctuation is evident regardless of whether engagement is examined at the level of specific language skills (Zhang & Gao, 2024) or at the overall classroom level (Vo et al., 2025).

While this body of research proffers valuable insights into the developmental trajectories of L2 learner engagement, thus informing policies and practices aimed at promoting learner engagement and effective L2 learning, it is limited in the sense that these trajectories are assumed to be generalizable to all participants. This is not always true, given that students experience intra‐individual changes in engagement over time, and there are inter‐individual differences associated with such changes (Vo et al., 2024). In other words, students do not necessarily follow a uniform growth trajectory in engagement: some demonstrate increases over time, others experience decreases, while still others remain stable. Assuming that the growth trajectory of L2 learner engagement is common to all participants overlooks the potential existence of unobserved but meaningful sub‐groups of L2 learners who might show a diverging pattern of growth from the overall sample and thus requires more tailored forms of support. Missing out on opportunities to identify such sub‐groups of L2 learners might prove costly as intervention and support programs risk not being directed toward those who need them most. A plausible reason for this limitation in prior research is that they rely on conventional approaches to modelling changes in L2 learner engagement (i.e., latent growth curve modelling, multilevel modelling), which assumes that individuals come from a single population for which a single set of growth parameters can be estimated. Therefore, to examine heterogeneity with respect to engagement trajectories within the L2 student population requires a methodological framework that relaxes the homogeneity assumption and allows for heterogeneities in growth parameters across unobserved sub‐populations (Jung & Wickrama, 2008). To address this critical research gap, I aim to uncover, through longitudinal growth mixture analyses, the classroom engagement trajectories of distinct groups of L2 learners over the course of a semester and examine how these trajectory groups are shaped by key L2 learning antecedents and, in turn, shape subsequent L2 learning outcomes.

## LITERATURE REVIEW

### L2 engagement

For the purpose of this study, I define L2 engagement as “the quality of L2 learners' participation in and interaction with teachers, peers, and learning activities within the dynamic context of the language classroom” (Vo et al., 2024) and seek to discover the developmental trajectory of L2 learner classroom engagement over an academic semester. Within this context, L2 learner classroom engagement is manifested in behaviour, cognitive and affective aspects. Behaviour engagement refers to the observable behaviours L2 students exhibit while participating in the language classroom such as time on task, voluntary involvement in speaking and interactional initiatives (Sang & Hiver, 2021). Cognitive engagement pertains to the mental processes while participating in language learning activities such as connecting existing and new knowledge, using language learning strategies commensurate with a given learning task or a willingness to push oneself beyond expectations (Vo et al., 2025). Finally, affective engagement is concerned with the positive (e.g., enjoyment, excitement) or negative (e.g., anxiety, boredom) feelings in relation to language learning activities or other socializers in the language classroom (Vo, 2024). Although these dimensions of L2 engagement have been found to be differentially associated with various antecedents and outcomes in the language learning process, genuine engagement relies on the simultaneous and effective coordination of all three, thereby offering a holistic picture of how L2 learners act, think and feel in the language classroom (Mercer, 2019).

### Longitudinal L2 engagement

Engagement is neither monolithic nor static. It evolves over time, with changes occurring at varying rates and in different forms. These characteristics have been empirically confirmed by a steady stream of research on longitudinal student engagement in the learning sciences and educational psychology more broadly (Salmela‐Aro et al., 2021). In their scoping review of a decade of research on longitudinal student engagement (2010–2020), Salmela‐Aro et al. (2021) noted that despite the field's exponential growth, the evidence base remained largely Western‐centric, highlighting the need for more research in non‐Western contexts. Although they did not address domain‐specific longitudinal engagement, we speculate that a similar imbalance exists, with the literature predominantly focusing on STEM‐related or general school engagement. In fact, the L2 learning domain is just beginning to understand the development of L2 learner engagement over time and the various growth trajectories that characterize such development, though such studies are few and far between. Some studies zoom in on the development of L2 student engagement within a language task, with engagement being measured at the timescale of minutes (Dao & Sato, 2021; Sulis, 2022; Sulis & Mercer, 2025). For example, Dao and Sato (2021) measured L2 student emotional engagement after every 5‐min interval over the course of a 15‐min communicative task using the experience sampling method. They found that positive emotional engagement increased significantly from the first to the second interval, then stabilized toward the end of the task. These increases were found to be positively associated with the amount of L2 production (i.e., word counts, turn counts) and the degree of collaboration (i.e., mutuality and collaboration).

Other studies look at the trajectories of L2 student engagement with a specific language skill during a lesson, a series of tasks or a course of study (Aubrey et al., 2020; Li & Jiang, 2024; Xiaosa & Ping, 2025; Zhang & Gao, 2024). In one such study, Zhang and Gao (2024) examined the dynamic changes in six undergraduate L2 students' engagement with peer feedback across three writing cycles in a writing course. They reported that student behavioural, cognitive and affective engagement with peer feedback dynamically and non‐linearly interacted with one another within and across tasks. In addition, these dynamic and evolving interactions were shaped by various individual and contextual factors, including language proficiency, learner beliefs, feedback accuracy and feedback features. Another line of research takes a more holistic approach to tracking L2 student engagement at the classroom level over an academic semester or school year (Vo et al., 2024, 2025; Zhou, Hiver, & Zheng, 2023). For example, Vo et al. (2025) measured L2 student classroom engagement and task value beliefs at three equidistant intervals over the course of a 15‐week semester and found that engagement and task value beliefs are dynamically and reciprocally related with each other at both inter‐ and intra‐individual levels.

Taken together, the growing body of longitudinal L2 engagement research to date has underscored the malleable, dynamic and constantly evolving nature of L2 student engagement regardless of the contexts in which engagement occurs and the time frames over which it is measured. This body of research, however, does not account for the fact that not all students follow the same engagement growth trajectories, nor are their growth trajectories influenced by covariates in the same ways. Prior studies either employ qualitative case studies or rely on variable‐centred longitudinal designs that preclude the identification of unobserved but meaningful sub‐groups of L2 students who might exhibit varied patterns of engagement growth. For example, although Vo et al. (2024), employing latent growth curve modelling, found both the mean and the variance of the engagement slope factor to be significant, demonstrating overall growth in engagement and interindividual differences in the rate of growth. This only implies that all students follow a similar growth pattern, with individual rates of growth varying around a common mean. This overlooks the possibility that individuals might be typed into distinct groups representing diverse growth patterns, with individual students' rates of growth varying around the mean of their own group. The identification of such distinct groups of students holds considerable potential for differentiated interventions as teachers and school leaders can not only design support programs for students' long‐term engagement but also provide the right kind of support for the right type of students. Person‐centred longitudinal analyses, such as growth mixture modelling (GMM), are specifically designed to account for longitudinal heterogeneity in the developmental trajectories of different learning outcomes such as L2 engagement (Wickrama et al., 2021). This is achieved by relaxing the homogeneity assumption of a common growth pattern, allowing different unobserved groups of students to emerge from the data, each having distinct intra‐individual growth trajectories and inter‐individual differences in the rates of growth (Jung & Wickrama, 2008). Therefore, GMM represents a more nuanced alternative to the conventional variable‐centred analyses of the development of L2 student classroom engagement, thereby capturing unique developmental perspectives that prior studies have been unable to address.

### Predictors of longitudinal L2 learner classroom engagement

For many scholars, engagement is a relational process, reflecting the quantity and quality of students' interactions with the activities and other social partners in the learning process. It is not inherently a characteristic of the learners themselves, but of their relationally mediated participation in learning opportunities structured through social interactions (Hofkens & Ruzek, 2019). Therefore, the classroom represents a developmental and relational context in which teachers serve as important attachment figures, and their relationships with students constitute a developmental asset that fosters growth (Hofkens & Pianta, 2022). This is particularly relevant in the context of learning English as a foreign language, given that language learning is inherently a transactional process and that the classroom serves as the primary setting for English learning because students have little opportunity to engage with the language beyond this context (Vo et al., 2025). Indeed, our review of the L2 engagement literature indicated that teachers are the most extensively researched classroom variable and that the teacher–student relationship has been consistently reported as a significant predictor of L2 student engagement across studies. Therefore, understanding the nature of this relationship and teacher practices that foster quality interactions in the classroom are key to supporting L2 student engagement growth. The Teaching Through Interaction (TTI) framework posits that teachers can structure quality classroom interactions through providing emotional support, effective classroom management and instructional support (Hamre et al., 2013; Pianta & Hamre, 2009). In this study, we focus on emotionally supportive interactions in the classroom as a predictor of longitudinal L2 student classroom engagement, given the salient role of teacher emotional support in supporting L2 student engagement reported in prior cross‐sectional studies.

Teacher emotional support refers to the organization of teacher–student interactions based on a genuine concern for and care about students as well as an interest in and respect for their points of view and perspectives (Hofkens & Pianta, 2022). Emotionally supportive interactions are demonstrated through positive emotional climate, teacher sensitivity to students' emotional needs and teacher regard for students' perspectives (Hofkens & Pianta, 2022; Pianta & Hamre, 2009). Positive emotional climate describes classrooms characterized by the presence of warm and caring interactions and the absence of punitive, agitated and humiliating interactions. Teacher sensitivity reflects teachers' awareness of, and responsiveness to cues about students' emotional needs, providing timely support to those who struggle emotionally in the classroom. Regard for students' perspectives involves valuing students' personal viewpoints and ideas, orienting learning and interactions to students' interests and motivation and incorporating their feedback in the instructional process. Research has suggested that students taught by teachers who are responsive to students' needs, respect students' viewpoints and perspectives and make students feel emotionally secure rather than pressured tend to show higher levels of engagement in the classroom (Havik & Westergård, 2020; Rimm‐Kaufman et al., 2015; Ruzek et al., 2016). In the L2 learning domain, teacher emotional support has also been found to be a critical antecedent of L2 student engagement in a growing body of research (Sulis & Philp, 2020; Zhou, Liu, & Guo, 2023). However, L2 studies that specifically examine emotional climate, teacher sensitivity and regard for students' perspectives as the multiple dimensions of an emotionally supportive interaction framework and how they shape the developmental trajectories of L2 student engagement in the classroom remain limited. In one exception, Vo et al. (2024) found that of the three emotionally supportive interaction components, only teacher sensitivity significantly predicted the interindividual differences in the rate of growth of L2 student engagement, suggesting that those who perceived teachers as emotionally responsive exhibited a greater rate of engagement growth over time. Building on Vo et al. (2024), this study investigates the extent to which emotional climate, teacher sensitivity and regard for students' perspectives influence the likelihood of L2 students being classified into distinct profiles characterized by different trajectories of engagement growth over an academic semester.

### Outcomes of longitudinal L2 learner classroom engagement

Different engagement trajectories might yield different outcomes for different groups of L2 learners. In this study, I focus on classroom affect as a long‐term outcome of L2 learner classroom engagement. Classroom affect refers to students' overall emotional experiences and feeling states in the classroom (Watson et al., 1988). In the emotion research literature, affect is considered an omnibus construct encompassing both moods and emotions and is typically differentiated into positive and negative dimensions (Linnenbrink, 2006). Positive classroom affect reflects students' pleasurable experiences with the classroom environment, including feelings of enjoyment, happiness and joy, whereas negative classroom affect refers to students' subjective distress in the classroom, demonstrated through a range of negative feelings such as fear, anxiety and anger (Jiang et al., 2018; Jiang & Konorova, 2023; Watson et al., 1988).

In the L2 learning domain, affect is often studied as discrete emotions tied specifically to L2 learning activities or L2 achievements, and it is often identified as a significant predictor of L2 engagement. For example, positive emotions such as enjoyment, hope and pride have been found to positively predict L2 student engagement (Derakhshan & Yin, 2024; Dewaele & Li, 2021; Zou et al., 2025), whereas negative emotions such as boredom and anxiety exert negative impacts (Ma & Xu, 2025; Wang & Hui, 2024; Zhao & Wang, 2024). These findings are consistent with the bulk of research evidence on the predictive effect of emotions on student engagement in the learning sciences and educational psychology more broadly (Linnenbrink, 2006; Pekrun & Linnenbrink‐Garcia, 2022). However, in this study, I position positive and negative classroom affect as long‐term outcomes of L2 classroom engagement trajectories for both theoretical and conceptual reasons.

Conceptually, positive and negative affect, when conceptualized as overall classroom experiences rather than discrete emotions directly linked to achievement activities or outcomes, can be considered indicators of students' subjective wellbeing (Datu & King, 2018; Jiang et al., 2018; Jiang & Konorova, 2023). Subjective wellbeing, defined as individuals' overall evaluation of contentment with their life, including both cognitive (i.e., life satisfaction) and affective evaluations (i.e., positive and negative affect), has been well‐established as a desirable educational outcome (Diener, 2006; OECD, 2013). Educational research and practice have demonstrated that engaged students tend to experience higher levels of life satisfaction and enjoyment and lower levels of depression or negative emotions, suggesting that positive and negative affect are immediate outcomes of student engagement (Datu & King, 2018; Wong et al., 2024). However, L2 research has seldom examined positive and negative affect as outcomes of L2 student engagement, nor considered them as developmental consequences of L2 engagement trajectories. Theoretically, although extensive research has established the effects of emotions on student engagement, scholars have also documented a reciprocal feedback loop in which the reverse effects of engagement on emotions are likewise evident (Pekrun & Linnenbrink‐Garcia, 2022). For example, Wang, Henry and Degol (2020) and Gladstone et al. (2022) argue that engaged students not only reap benefits from their engagement experiences in the classroom, but also develop affective reactions to these experiences, which in turn feed into their ongoing self‐appraisals and engagement. Here, I adopt a developmental perspective of L2 student classroom engagement and argue that as student engagement deepens over time, L2 students cumulatively develop positive affective evaluation of the overall classroom experiences, whereas declining engagement is more likely to foster negative affective experiences.

### The present study

In this study, I aim to uncover distinct profiles of L2 students who exhibit diverse growth patterns of L2 classroom engagement. I further examine whether the three dimensions of teacher emotional support, including emotional climate, teacher sensitivity and regard for students' perspectives, predict students' likelihood of profile membership and how these profiles shape students' positive and negative classroom affect. The study is, therefore, guided by the following research questions (see Figure 1):

1. What profiles characterize the diverse developmental trajectories of L2 student classroom engagement over the course of a semester?
  Based on longitudinal evidence suggesting meaningful within‐person variabilities (see Section 2.2), I expect to identify multiple distinct developmental trajectories of L2 student engagement rather than a single homogeneous pattern. However, I do not advance specific hypotheses regarding the configuration of growth parameters within these profiles because the identification of trajectory shapes in this person‐centred framework is inherently exploratory.
2. How does teacher emotional support measured at the beginning of the semester predict the likelihood of students being classified into those profiles?
  Based on prior cross‐sectional research evidence linking teacher emotional support with L2 student engagement (see Section 2.3), I expect that higher perceived teacher emotional support at the beginning of the semester will increase students' likelihood of membership in the profiles characterized by higher or improving engagement trajectories.
3. How do students in these profiles differ in terms of their negative and positive classroom affect measured at the end of the semester?
  Consistent with theoretical perspectives that view affective evaluation of classroom environment as a desirable outcome of engagement (see Section 2.4), I anticipate that students in profiles characterized by higher or increasing engagement trajectories will report higher positive classroom affect and lower negative classroom affect.

**FIGURE 1:**
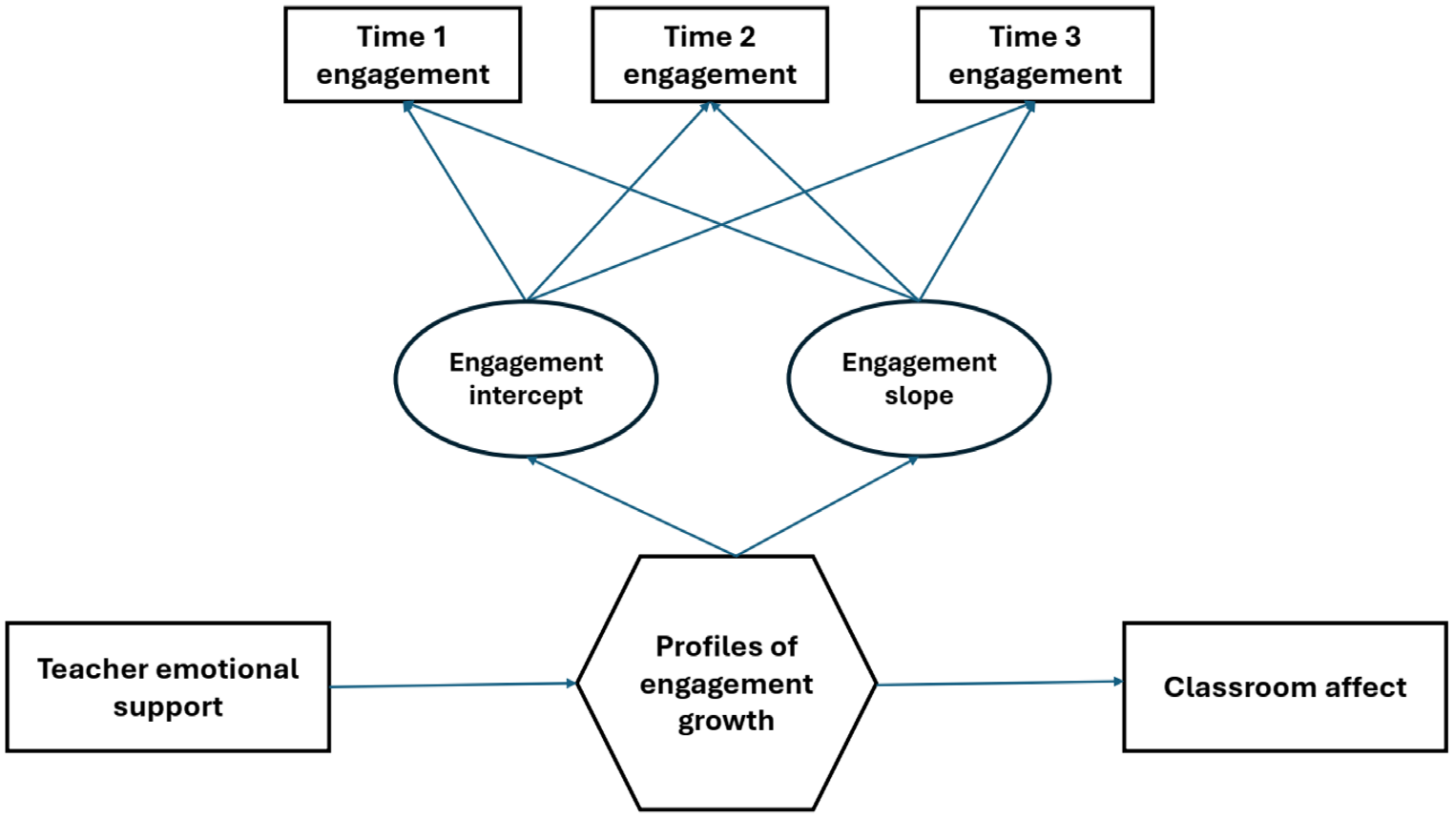
The conceptual model.

## MATERIALS AND METHODS

### Participants

Participants were recruited from the integrated listening and speaking module offered to first‐year English as a foreign language (EFL) students at a university in Vietnam. The module was delivered over a 10‐week term with 4.5 contact hours per week. The Bachelor of English language Program in which the participants were enrolled is a 3.5‐year program, which requires students to achieve the equivalent B1 level of English proficiency, as defined by the Common European Framework of Reference, by the end of the first year. The module was designed to develop students' English listening and speaking skills by integrating a series of task‐based communicative activities into each lesson. Students were involved in a variety of learning activities including pair work, groupwork, role‐play, presentations, classroom debates and project‐based group assignments. Instructions were delivered by 8 lecturers (2 males and 6 females), each holding a master's degree in teaching English to Speakers of Other Languages (TESOL) and at least five years of teaching experience at the tertiary level. All lecturers follow a consistent pedagogical framework, emphasizing communicative language teaching. English was the main medium of instruction, with occasional use of Vietnamese, the mother tongue of all students, to facilitate comprehension and engagement.

Data were collected during week 2, week 5 and week 8 over the 10‐week period of the module. Prior to completing the questionnaire, students received an information sheet and informed consent package outlining the purpose of the study, their role in it, the voluntary and anonymous nature of their participation and their right to withdraw at any time. A total of 497 students (M_age_ = 18.54; N_female_ = 432) completed the questionnaire at wave 1, 449 students completed at wave 2 and 388 completed at wave 3, representing an attrition rate of 9.7% and 13.6% between successive waves. Little's MCAR conducted on the study variables across all three waves yielded a non‐significant *p* value (*χ* ^2^ (14) = 12.136, *p* = .595), suggesting the randomness of the missing data mechanism.

### Instruments

L2 student classroom engagement was measured by a brief three‐item scale adapted from Zhou and Hiver (2022). These three items asked students to report on the levels of their behavioural, cognitive and affective participation in the language classroom. An example item is: “Think about the different ways you can actively participate in an English class (e.g., raising your hand & asking questions; consulting your peers & instructors; paying attention to the teacher's instructions, etc.). How would you rate your level of participation in these ways during your English class?”.

L2 students' perceived teacher emotional support was assessed by an 8‐item scale adapted from Wang, Hofkens and Ye (2020). The scale includes two items assessing emotional climate (e.g., *My English teacher says nice things to me*.), three items assessing teacher sensitivity (e.g., *My English teacher understands how I feel about things in class*.) and three items assessing teacher regard for students' perspectives (e.g., *My English teacher lets me know that if I do not agree with him/her, it is important that I express my disagreement*.).

Positive and negative classroom affect were measured by a 7‐item scale adapted from Jiang et al. (2018). Positive affect was gauged by three items (e.g., *Most of the time, being in this class puts me in a good mood*), while negative affect was gauged by four items (e.g., *This English class often makes me feel bad*.).

These three scales have been validated in previous studies and have been found to yield adequate psychometric properties (see, for example, Jiang et al., 2018; Vo et al., 2024, 2025; Wang, Hofkens, & Ye, 2020).

### Procedure

Students responded to the questionnaires on a five‐point Likert‐type scale, with higher values indicating higher levels of each construct. Students completed the L2 classroom engagement questionnaire three times over the semester (at week 2, week 5 and week 8), the perceived teacher emotional support questionnaire at the first time point (week 2) and the classroom affect questionnaire at the third time point (week 8). Perceived emotional support was measured in Week 2 because the construct is conceptualised in this study as an antecedent predictor of students' engagement trajectories. Early assessment establishes temporal ordering – the predictor (Week 2) precedes the repeated outcome measurements used to model engagement over time – thereby strengthening causal inference for the hypothesized directional effect and allowing insights into how early exposure to teacher emotional support shapes L2 learners' engagement growth trajectories over the semester. I acknowledge that assessments later in the semester may reflect accumulated experience with the teacher, and I noted this as a limitation and a recommendation for future work to include repeated measurements of support. Similarly, because my focus was on classroom affect as an outcome of the developmental trajectories of L2 engagement, this construct was measured at week 8. Assessing this construct at the end of the study reflects the intended temporal ordering, enabling it to capture students' overall positive or negative affective experiences as a result of their unfolding engagement trajectories.

Item wording was adjusted to suit the specific context of English as a foreign language learning in this study. For example, the item “Math class often makes me feel bad” in the original negative classroom affect scale was modified as “This English class often makes me feel bad.” All questionnaire items were translated into Vietnamese to facilitate students' response. The translated version was first piloted with a group of students with similar backgrounds to those in the main study to verify the suitability, comprehensibility and completeness of the items. Any concerns were resolved in a moderation session before the final questionnaire was administered in the main study. The psychometric properties of these three scales are presented in Section 4.1.

### Data analysis

Data analysis was conducted in three steps. In step one, I performed confirmatory factor analyses to examine the psychometric properties of the teacher emotional support and classroom affect scales as well as the longitudinal measurement invariance of the L2 student classroom engagement scale. This is done by sequentially estimating and comparing a series of hierarchically nested models: a configural invariance model with all parameters freely estimated (baseline model), a metric invariance model with equality constraints on factor loadings (weak invariance), a scalar invariance model with equality constraints on factor loadings and item intercepts (strong invariance) and a residual invariance model with equality constraints on factor loadings, item intercepts and residual variances (strict invariance) (Wickrama et al., 2021). Assessment of model fit was based on typical statistical fit indices recommended in the literature, including the Comparative Fit Index (CFI > .95), Tucker–Lewis Index (TLI > .95), Root Mean Square Error of Approximation (RMSEA < .06), Standardized Root Mean Square Residuals (SRMR < .08) (Hu & Bentler, 1999). To compare alternative models of longitudinal invariance, I followed Cheung and Rensvold (2002) recommendations to examine changes in CFI (∆CFI) and RMSEA (∆RMSEA) values. Changes exceeding .01 for CFI and .015 for RMSEA suggest that imposing additional equality constraints reduces model fit, and thus, the model with fewer constraints should be preferred.

In step two, I first estimated a linear latent growth curve model (LGCM) to understand the overall growth trajectory of L2 student classroom engagement over time. This growth trajectory was captured by two continuous latent factors: an intercept factor that represents the initial level of L2 student classroom engagement at the beginning of the semester, and a slope factor that represents the intra‐individual changes in engagement over the course of the semester. These two factors were represented by the same three indicators, which reflect the aggregated scores of L2 student classroom engagement across the three time points. The loadings of these indicators on the intercept were fixed at 1, whereas their loadings on the slope factor were fixed at 0, 1 and 2, respectively, consistent with the default specification of a linear LGCM (Wickrama et al., 2021).

Next, I built on this baseline model to conduct growth mixture modelling (GMM). Linear GMMs with solutions ranging from one to five latent trajectories of L2 student classroom engagement were estimated because models with six or more profiles did not converge on a proper solution. To avoid convergence on local solutions, analyses were conducted using 7000 random sets of start values, 500 iterations for each random start and 250 best solutions retained for final stage optimization (Hipp & Bauer, 2006). I relied on the default specifications in Mplus, which freely estimate the intercept and slope means, but constrain the variances, covariances and residual variances to be invariant across profiles. To determine the most optimum profile solution, I relied on key goodness of fit indices including the Akaike Information Criterion (AIC), the Consistent AIC (CAIC), the Bayesian Information Criterion (BIC), the sample size adjusted BIC (ABIC), the Vuong‐Lo–Mendell–Rubin likelihood ration test (VLMR‐LRT) and the bootstrapped likelihood ratio test (BLRT). AIC, CAIC, BIC and ABIC are relative fit information criteria with lower values indicating better model fit (van de Schoot et al., 2017). VLMR‐LRT and BLRT are likelihood ration tests with significant values (*p* < .05), suggesting the superiority of the k‐profile solution against the k‐1 profile solution (Masyn, 2013; Nylund‐Gibson & Choi, 2018). I also checked the entropy value and the average posterior class probabilities (AvePPk) as measures of accuracy and reliability of the profile enumeration process. Entropy values approaching 1 and AvePPk higher than .80 are desirable (Spurk et al., 2020; van de Schoot et al., 2017).

In the final step, I incorporated covariates into the best‐fitting profile model to examine the predictive effects of teacher emotional support on students' likelihood of profile membership and to test whether the profiles differed in students' classroom affect. Profile membership was predicted using multinomial logistic regression via the auxiliary R3STEP function, whereas profile differences in classroom affect were evaluated using the Wald chi‐square equality tests of means implemented through the auxiliary DCON function in Mplus (Wickrama et al., 2021).

All analyses were executed in Mplus version 8.11, using the robust maximum likelihood estimation method, which generates parameter estimates, standard errors and model fit indices that are robust to potential non‐normality in the data. To address the small amount of missing data, I relied on the automatic implementation of the full information maximum likelihood procedures (FILM) in Mplus. FILM produces unbiased parameter estimates by using all available information in the data rather than using listwise deletion strategies or the computationally intensive multiple imputation procedures (Enders & Bandalos, 2001).

## FINDINGS

### Preliminary analyses

Mean, standard deviation and correlations among the study constructs are presented in Table A1. Confirmatory factor analyses yielded adequate fit indices for the teacher emotional support (*χ* ^2^ (17) = 56.442, *p* < .001; CFI = .964; TLI = .940; RMSEA = .068; SRMR = .029) and classroom affect (*χ* ^2^ (13) = 35.484, *p* < .001; CFI = .974; TLI = .957; RMSEA = .067; SRMR = .016) scales. All standardized factor loadings are significant and substantial, ranging from .624 to .885 for teacher emotional support and .670 to .922 for classroom affect (Table A2). Composite reliability measures are acceptable with Omega McDonald values ranging from .711 for emotional climate to .938 for negative affect. Results of longitudinal measurement invariance tests for the L2 student classroom engagement scale (Table A3) suggest that imposing additional equality constraints in the metric and scalar invariance models did not change CFI (.100) and RMSEA (.000) values from the baseline model. When constraining factor loadings, item intercepts and residual variances in the residual invariance model, CFI decreased by .002 and RMSEA increased by .014, both of which are still within the limit recommended by Cheung and Rensvold (2002). These results indicate that the L2 classroom engagement scale remained strictly invariant across the three time points, thus enabling the meaningful interpretation of changes in student engagement over time.

### GMM

The linear latent growth curve model represents a good fit to the data (*χ* ^2^ (1) = 6.985, *p* < .01; CFI = .982; TLI = .945; SRMR = .025). The means of the intercept (Est. = 4.141, *p* < .001) and slope factors (Est. = .040, *p* < .05) were statistically significant, suggesting a linear growth trajectory with an increasing, yet modest rate of growth for L2 student classroom engagement over the semester. To examine whether heterogeneous subpopulations of students underlie this overall pattern of growth, I conducted linear growth mixture modeling.

Table 1 presents statistical fit indices for GMMs with solutions ranging from 1 to 5 profiles. Across all profile solutions, only the 4‐profile solution yielded a statistically significant VLMR‐LRT test, suggesting that the 4‐profile solution improved model fit over the 3‐profile solution. The values of AIC, CAIC, BIC and ABIC kept decreasing as the number of profiles increased, with the largest drop in values occurring in the 4‐profile solution. The elbow plot of the information criteria (Figure 2) reveals a marked decline in all information criteria from the three‐ to four‐profile solutions, after which the values levelled off, indicating little improvement in model fit from the five‐profile solution onward. Based solely on statistical fit indices, the four‐profile solution represents the best fit. Upon closer examination of the makeup of the three‐ and four‐profile solutions, I found all the resultant profiles to be theoretically and substantively meaningful. The smallest profile in the three‐profile solution includes 52 participants (12% of the sample), whereas the smallest profile in the four‐profile solution includes 11 participants (2.2% of the sample), which is below the recommended threshold of 5%. However, given that the purpose of GMMs is to identify meaningful sub‐groups of individuals who represent a diverging pattern of growth from the overall sample, and that the additional profile in the four‐profile solution, albeit small in size, shows a unique but concerning pattern of growth that should not be overlooked, I decided to retain the four‐profile solution as the best fitting model. This decision was consolidated by the high entropy (.846) and AvePPk values (.884–.938), suggesting adequate reliability and accuracy of the profile enumeration process.

**TABLE 1:** Statistical fit indices for the growth mixture models.

No. profile	LL	No. fp	Scaling	AIC	CAIC	BIC	ABIC	Entropy	VLMR‐LRT
1 profile	−1199.984	8	1.4552	2415.969	2457.637	2449.637	2424.245	N/A	
2 profiles	−1186.725	11	1.4118	2395.449	2452.744	2441.744	2406.829	.906	*p* = .107
3 profiles	−1164.777	14	1.9244	2357.554	2430.474	2416.474	2372.038	.746	*p* = .526
4 profiles	−1108.024	17	1.2466	2250.048	2338.594	2321.594	2267.635	.846	*p* < .001
5 profiles	−1096.312	20	1.2740	2232.624	2336.795	2316.795	2253.315	.859	*p* = .130

**FIGURE 2:**
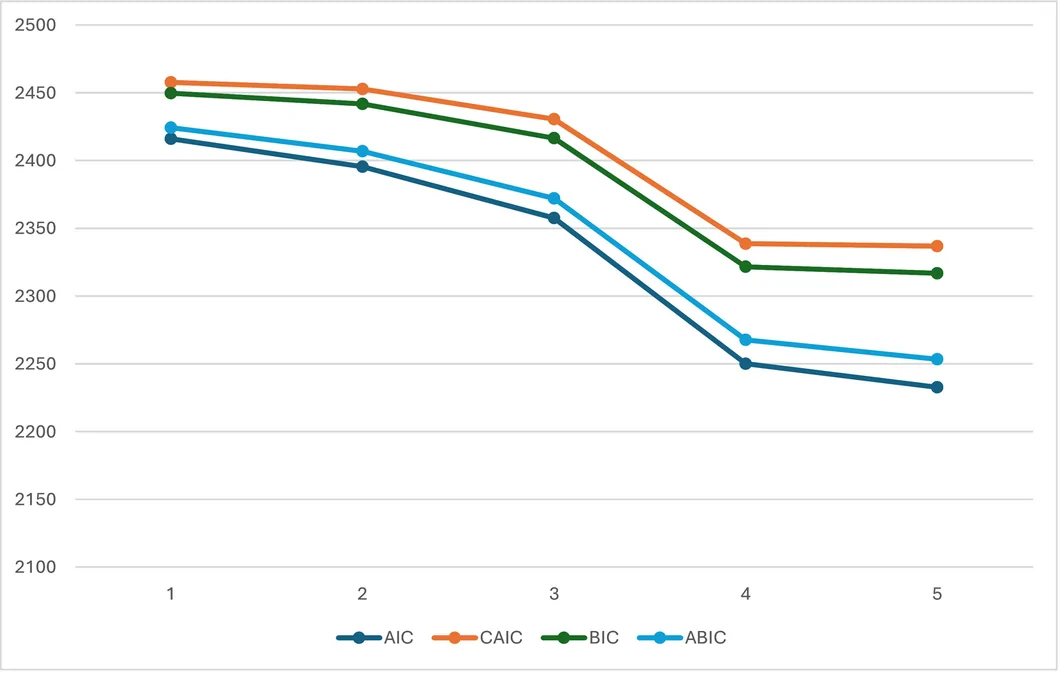
Elbow plot of statistical fit indices. ABIC, the sample size adjusted BIC; AIC, Akaike Information Criterion; BIC, Bayesian Information Criterion; BLRT, bootstrapped likelihood ratio test; CAIC, Consistent AIC; VLMR‐LRT, Vuong‐Lo–Mendell–Rubin likelihood ratio test; x‐axis, solutions ranging from 1 to 5 profiles; y‐axis, values of the fit indices.

Table 2 presents the growth parameters of the four‐profile solution. Profile one includes 190 students (38.2% of the sample) who show a high initial level of engagement, which then slightly increases toward the end of the semester. This profile is thus labelled the *high initial and slight increase* profile. Profile two represents 47.8% of the sample (*N* = 238) and is characterized by a high initial level of engagement, which remains stable until the end of the semester. This profile is labelled *high initial and stable* profile. Profile three constitutes 11.6% of the sample (*N* = 58) and demonstrates a moderate level of engagement, which then slightly decreases over time. This profile is labelled *moderate initial and slight decrease* profile. The last profile includes 11 students (2.4% of the sample) who show a rather concerning pattern: moderate level of engagement with a steep decrease over time. This profile is therefore labelled *moderate initial and steep decrease* profile. A graphical illustration of these profiles is presented in Appendix 2; Figures A1, A2, A3, A4.

**TABLE 2:** Profile size and parameter estimates for the four‐profile solution.

	Profile 1—High and slight increase	Profile 2—High and stable	Profile 3—Moderate and slight decrease	Profile 4—Moderate and steep decrease
Profile size	190	238	58	11
Intercept mean	4.391**	4.103**	3.647**	3.579**
Slope mean	.259**	−.023	−.222**	−.694**
Intercept variance	.146**	.146**	.146**	.146**
Slope variance	.033**	.033**	.033**	.033**

** Parameters are significant at *p* < .001.

### Predictors of profile membership

Table 3 presents results of multinomial logistic regression analyses testing the hypothesized effect of teacher emotional support on L2 students' likelihood of profile membership. Among the three components of teacher emotional support, only teacher sensitivity significantly predicted students' likelihood of profile membership (Profile 1 vs. Profile 2: OR [95% CI] = 3.872 [1.943, 7.716]; Profile 1 vs. Profile 3: OR [95% CI] = 7.054 [3.150, 15.795]; Profile 1 vs. Profile 4: OR [95% CI] = 15.591 [2.611, 93.097]). More specifically, higher perceived teacher sensitivity increased the likelihood of students being classified into profile one (the *high initial–slight increase* profile) compared to all the other profiles. However, teacher sensitivity did not significantly affect students' likelihood of membership in profiles two, three and four.

**TABLE 3:** Results of multinomial logistic regressions.

Predictor	Profile 1 vs. profile 2		Profile 1 vs. profile 3		Profile 1 vs. profile 4	
	Coef. (SE)	OR [95% CI]	Coef. (SE)	OR [95% CI]	Coef. (SE)	OR [95% CI]
Emotional climate	−.173 (.274)	.841 [.492, 1.437]	−.224 (.343)	.799 [.408, 1.565]	−.895 (.798)	.409 [.085, 1.953]
Teacher sensitivity	1.354 (.352)**	3.872 [1.943, 7.716]	1.954 (.411)**	7.054 [3.150, 15.795]	2.747 (.912)**	15.591 [2.611, 93.097]
Regard	.072 (.266)	1.075 [.639, 1.808]	−.357 (.390)	.700 [.326, 1.502]	.226 (.507)	1.254 [.464, 3.389]

	Profile 2 vs. profile 3		Profile 2 vs. profile 4		Profile 3 vs. profile 4	
	Coef. (SE)	OR [95% CI]	Coef. (SE)	OR [95% CI]	Coef. (SE)	OR [95% CI]
Emotional climate	−.051 (.287)	.950 [.541, 1.668]	−.722 (.762)	.486 [.109, 2.163]	−.671 (.788)	.511 [.109, 2.395]
Teacher sensitivity	.600 (.356)	1.822 [.907, 3.659]	1.393 (.870)	4.026 [.732, 22.142]	.793 (.936)	2.210 [.353, 13.850]
Regard	−.428 (.384)	.652 [.307, 1.384]	.154 (.487)	1.167 [.450, 3.029]	.583 (.568)	1.791 [.588, 5.457]

** Parameters are significant at *p* < .01.

### Outcomes of profile membership

Table 4 presents the estimated means of positive classroom affect and negative classroom affect along with the Wald Chi‐square equality tests of means across the four profiles. The results revealed statistically significant differences in positive classroom affect among profiles one, two and three. Students in profile one reported higher levels of positive classroom affect than those in profile two, who in turn reported higher levels than those in profile three (Profile 1: M = 4.293, Profile 2: M = 3.829, Profile 3: M = 3.456, Profile 4: M = 3.203). No significant differences were found for profiles three and four. In terms of negative affect, students in profile three reported significantly higher levels than those in profiles one and two (Profile 1: M = 2.024, Profile 2: M = 2.114, Profile 3: M = 2.494, Profile 4: M = 2.817). No other group differences were observed.

**TABLE 4:** Means and pairwise comparisons between the four profiles.

Outcome	Profile 1	Profile 2	Profile 3	Profile 4	Summary of significant differences
	M (SE)	M (SE)	M (SE)	M (SE)	
Positive classroom affect	4.293 (.058)	3.829 (.059)	3.456 (.108)	3.203 (.236)	1 > 2 > 3 = 4
Negative classroom affect	2.024 (.084)	2.114 (.081)	2.494 (.174)	2.817 (.418)	1 = 2 < 3 = 4

## DISCUSSION

This study contributes to the growing body of longitudinal L2 engagement research by uncovering heterogeneous subpopulations of L2 students who exhibit diverse trajectories of engagement over the course of an academic semester. Building on findings from prior cross‐sectional studies, the study further examines whether teacher emotional support measured at the beginning of the semester predicts the likelihood of students being in the different profiles of engagement trajectories as well as whether these profiles differ in the levels of positive and negative classroom affect assessed at the end of the semester.

### Profiles of L2 student classroom engagement trajectory

GMM revealed four different groups of students with distinct patterns of L2 classroom engagement growth: a *high initial and slight increase* group (38.2%), a *high initial and stable* group (47.8%), a *moderate initial and slight decrease* group (11.6%) and a *moderate initial and steep* decrease group (2.4%). Participants in this study were first‐year university students who had just experienced a transition period from high school to university settings. This period is known to present freshmen with academic challenges associated with changes in teaching, learning and classroom structure as well as demanding academic tasks, unfamiliar learning situations and more autonomy, but higher academic expectations (Gillet et al., 2019). These changes might affect how students experience the L2 classroom environment and underlie the development of various engagement trajectories specific to distinct groups of students. Consistent with the results of the initial latent growth curve modelling and with prior person‐centred longitudinal student engagement in school contexts (Archambault & Dupéré, 2017; Zhen et al., 2020), I identified a stable trajectory group and a slightly increasing trajectory group that together constitute 80% of the total sample. The observation that a large proportion of L2 students showed a stable trajectory of classroom engagement with a tendency to slightly increase toward the end of the semester could be explained by students' increasing familiarity with and adaptation to the classroom context. At the timescales of weeks or months typical of an academic semester, students gradually develop greater familiarity with the learning content and assessment requirements, build rapport with other social partners such as teachers and peers and improve their language ability to a level that allows them to continuously participate in and actively contribute to the ongoing flow of the L2 classroom discourse (Vo et al., 2024). These experiences enable students to maintain at least a stable level of engagement in the classroom, and those who cumulatively develop positive self‐appraisals of these experiences might gain momentum to engage at higher levels over time (Wang, Henry, & Degol, 2020). Beyond the dominant profiles of stability and slight increase, I also identified two profiles characterized by declining engagement: one marked by a modest decrease and the other by a pronounced decrease. These profiles, though small in size (20% of the total sample), present worrisome engagement trajectories that would have remained hidden within variable‐centred longitudinal analyses. In contrast to students in the other profiles, those in the declining engagement profiles might have developed maladaptive learning experiences that undermined their adaptability and hindered their engagement trajectory. Such maladaptive experiences might have been caused by a perceived mismatch between students' expectations and initial experiences at university, thus impeding their adjustment to the new learning environment (Hoi, 2023). Although these profiles reflect engagement trajectories that diverge from the overall sample, they demonstrate the value of a person‐centred longitudinal approach in pinpointing small groups of L2 students who require targeted support during their first semester at university. One way to optimize such support is to further examine how the different profiles of L2 engagement trajectories shape and are shaped by classroom dynamics.

### Predictors of profile membership

Results of multinomial logistic regression indicated that out of the three dimensions of teacher emotional support including emotional climate, teacher sensitivity and regard for students' perspective, only teacher sensitivity significantly predicted the likelihood of students being classified into the four engagement trajectory profiles. More precisely, students who perceived teachers as being more attentive and responsive to their emotional needs were more likely to belong to the *high initial–slight increase* profile than to all other profiles. This finding suggests that teacher sensitivity not only shapes how L2 student classroom engagement develops over time as was found in previous studies (Alrabai & Algazzaz, 2024, 2025; Vo et al., 2024), but also determines for whom such diverse developmental trajectories unfold. Previous studies support this finding, suggesting that teachers who accurately read students' emotional cues such as anxiety, sadness or boredom and identify those struggling emotionally and academically in class are more likely to adjust their instructional approaches and provide timely feedback. Such responsiveness helps students rebound from negative experiences, cultivate positive ones and sustain meaningful engagement in the classroom (Aldrup et al., 2020; Jennings & Greenberg, 2009).

### Outcomes of profile membership

Analyses of positive and negative classroom affect as outcomes of the different L2 engagement trajectory profiles suggest that the levels of positive classroom affect were highest in the *slight increase* profile, followed by the *stable* profile and lowest in the *slight decrease* profile. On the other hand, negative classroom affect was higher in the *slight decrease* profile than in the *slight increase* and the *stable* profile. These results support earlier studies in school contexts that found student engagement to be a positive predictor of positive affect and a negative predictor of negative affect, suggesting that engaged students are more likely to experience life satisfaction and positive emotions and less prone to negative classroom experiences (Datu & King, 2018; Wong et al., 2024). This study is among the first to examine classroom affect as outcomes of developmental L2 student classroom engagement from a person‐centred perspective, highlighting that as L2 students vary in their profiles of classroom engagement trajectories, so too does their positive and negative classroom affect as a function of engagement growth. In this respect, students in the slight decrease engagement profile represent a prime target for engagement support since they show the lowest levels of positive affect and the highest levels of negative affect.

## LIMITATIONS AND PRACTICAL IMPLICATIONS

Despite important findings yielded through a person‐centred longitudinal approach, this study has several limitations. First, this study assumes that L2 students follow a linear trajectory of classroom engagement. This is because engagement in this study was measured only at three time points over the semester, while the optimal estimation of non‐linear trajectories of engagement (e.g., quadratic or cubic growths) normally requires at least four data waves. Future studies might need to consider a longitudinal design that captures engagement at multiple time points to more comprehensively explore the various non‐linear growth functions and simultaneously to identify various hidden groups of students with both linear and non‐linear trajectories of engagement growth. Second, this study solely relies on students' self‐report data, which might be subject to social desirability biases. Triangulation of multiple data sources and multiple informants such as teacher ratings, observational measures and administrative data would increase the validity and reliability of the obtained results in future research. Finally, while the results of this study imply that L2 student classroom engagement, teacher sensitivity and classroom affect might reinforce one another over time, a thorough examination of the joint development of these constructs and the distinct groups of students characterized by these parallel processes are beyond the scope of this study. This represents an important research gap that future studies will need to address to gain a more complete understanding of the developmental heterogeneity of student engagement that emerges out of the dynamics of the language classroom.

This study demonstrates that being aware of the interindividual differences in L2 student engagement trajectories during the transition from school to university is crucial, given their potential influence on students' developmental functioning. Too often, teachers prioritize the overall classroom experiences while overlooking smaller, yet important groups of students who are more vulnerable to disengagement and subsequent maladaptive experiences. For L2 students who are in the early stages of adapting to a new learning environment and unfamiliar academic structures, the ways teachers show emotional support are of utmost importance. Being able to identify students who are struggling both emotionally and academically, and who are in need of an emotional boost, enables teachers to adjust their instructional and communication approaches in ways that nurture a secure teacher‐student relationship, thereby supporting their adaptation process. Therefore, in addition to professional development programs designed to enhance teacher instructional quality, those focused on strengthening teachers' emotional intelligence and emotional support strategies are equally crucial, as they equip teachers with the necessary skills and tools needed to support students effectively in the contemporary language classrooms.

## AUTHOR CONTRIBUTIONS

**Hoi Vo:** Conceptualization; investigation; writing – original draft; methodology; validation; visualization; writing – review and editing; software; formal analysis; project administration; data curation; supervision; resources.

## CONFLICT OF INTEREST STATEMENT

The author declares that there is no conflict of interest.

## Figures and Tables

**FIGURE A1:**
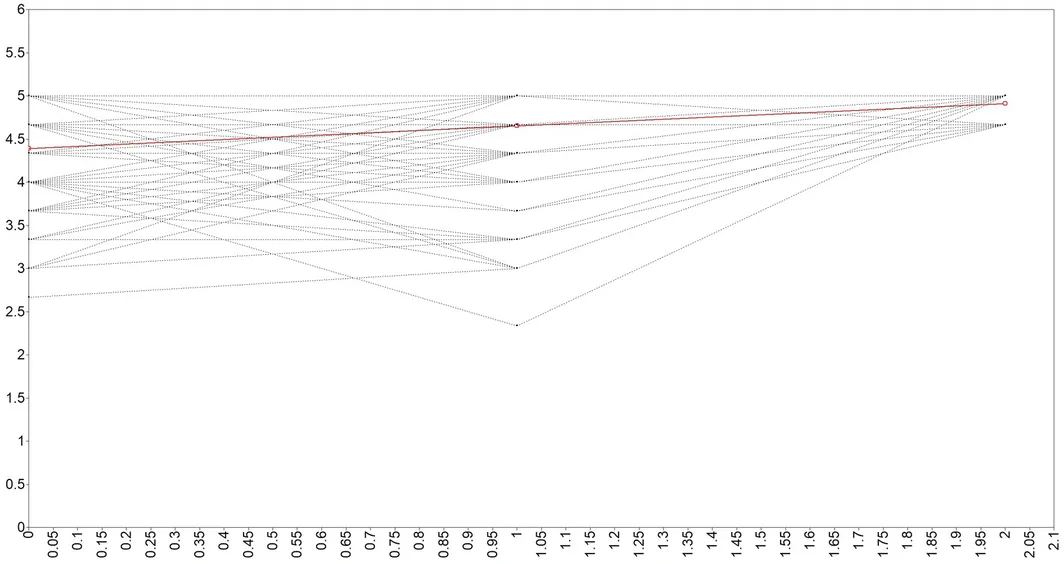
Profile 1—High initial and slight increase.

**FIGURE A2:**
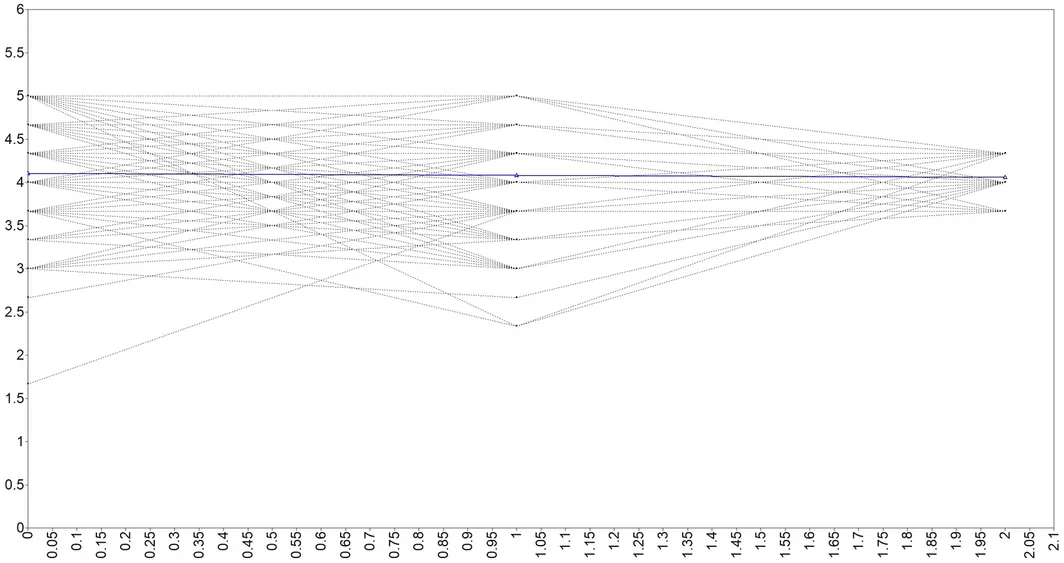
Profile 2—High initial–stable.

**FIGURE A3:**
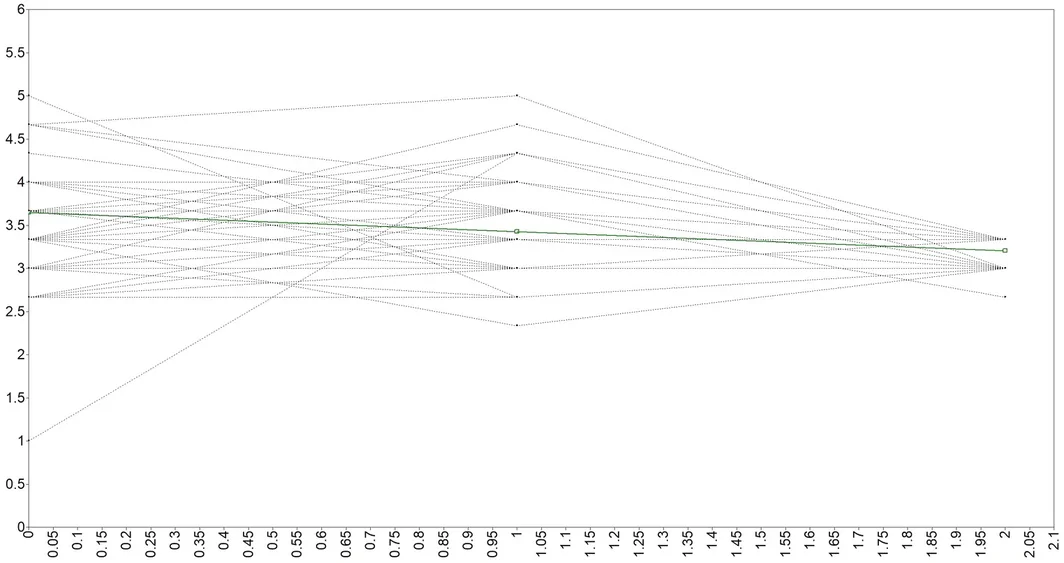
Profile 3—Moderate initial and slight decrease.

**FIGURE A4:**
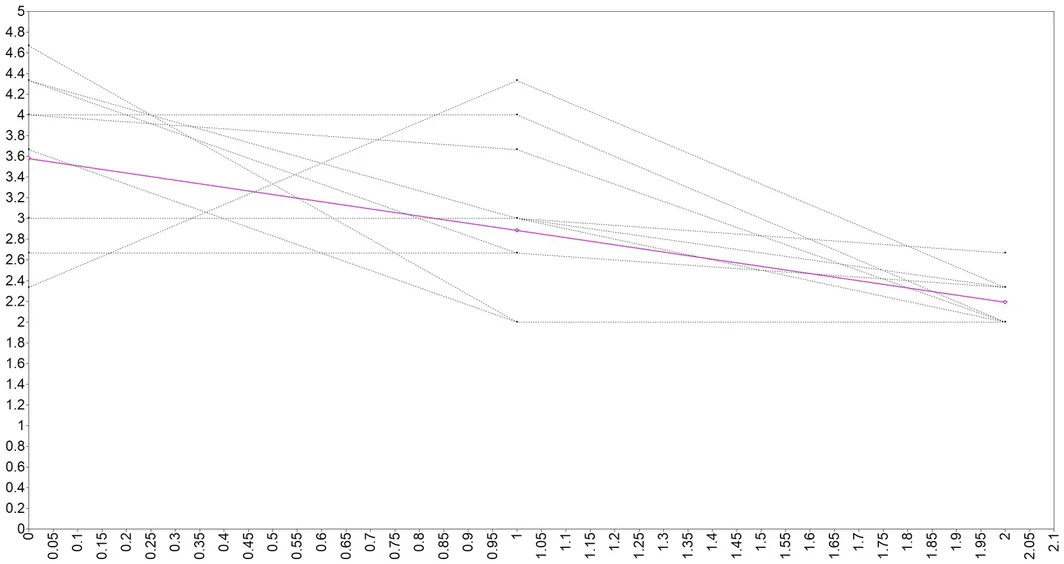
Profile 4—Moderate initial and steep decrease.

## Data Availability

The data that support the findings of this study are available on request from the corresponding author. The data are not publicly available due to privacy or ethical restrictions.

## APPENDIX 1

**TABLE A1:** Descriptive statistics.

	1	2	3	4	5	6	7	8
1. T1 engagement	1							
2. T2 engagement	.473**	1						
3. T3 engagement	.471**	.605**	1					
4. Positive affect	.224**	.368**	.392**	1				
5. Negative affect	−.157**	−.240**	−.165**	−.170**	1			
6. Emotional climate	.126**	.192**	.188**	.245**	−.154**	1		
7. Teacher Sensitivity	.238**	.320**	.344**	.425**	−.199**	.687**	1	
8. Regard	.153**	.245**	.221**	.318**	−.179**	.657**	.718**	1
Mean	4.164	4.140	4.236	3.947	2.145	4.619	4.416	4.466
SD	.622	.646	.688	.820	1.105	.678	.673	.721

** Correlations significant at *p* < .01.

**TABLE A2:** Standardized factor loadings for the scales.

Construct	Item	Factor loading (β)	Significant value
Classroom affect			
Positive affect (ω = .811)	Positive 1	.738	*p* < .001
	Positive 2	.670	*p* < .001
	Positive 3	.884	*p* < .001
Negative affect (ω = .938)	Negative 1	.830	*p* < .001
	Negative 2	.910	*p* < .001
	Negative 3	.922	*p* < .001
	Negative 4	.896	*p* < .001
Teacher emotional support			
Emotional climate (ω = .711)	Emotional 1	.633	*p* < .001
	Emotional 2	.808	*p* < .001
Sensitivity (ω = .792)	Sensitivity 1	.849	*p* < .001
	Sensitivity 2	.624	*p* < .001
	Sensitivity 3	.761	*p* < .001
Regards for students (ω = .870)	Regards 1	.841	*p* < .001
	Regards 2	.885	*p* < .001
	Regards 3	.763	*p* < .001

**TABLE A3:** Longitudinal measurement invariance of L2 classroom engagement.

	CFI	ΔCFI	RMSEA	∆RMSEA
Baseline model	1.00		.00	
Weak invariance model	1.00	.00	.00	.00
Strong invariance model	1.00	.00	.00	.00
Strict invariance model	.998	.002	.014	.014
